# Supplementary figures and images for: Transgenic LRRK2R1441G rats–a model for Parkinson disease?
Source: PeerJ. 2015 May 12;3:e945. doi: 10.7717/peerj.945 (PMC4435452; doi:10.7717/peerj.945)

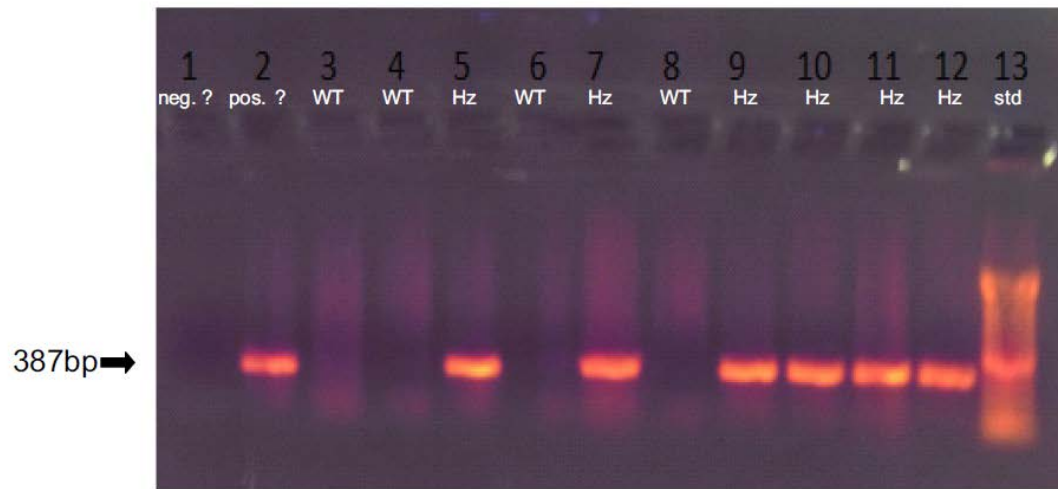

Supplement: Supplemental Information 2 — Genotype results show strong positive bands at 387 bp, indicating genomic presence of the human LRRK2 transgene. [file peerj-03-945-s002.pdf]
